# Hormonal influence: unraveling the impact of sex hormones on vascular smooth muscle cells

**DOI:** 10.1186/s40659-024-00542-w

**Published:** 2024-09-04

**Authors:** Keran Jia, Xin Luo, Jingyan Yi, Chunxiang Zhang

**Affiliations:** 1 Department of Medical Cell Biology and Genetics, School of Basic Medical Sciences, Basic Medicine Research Innovation Center for Cardiometabolic Diseases, Ministry of Education, Southwest Medical University, Luzhou, Sichuan 646000 China; 2 Department of Pharmacology, School of Pharmacy, Southwest Medical University, Luzhou, Sichuan 646000 China; 3 Department of Cardiology, The Affiliated Hospital, Basic Medicine Research Innovation Center for Cardiometabolic Diseases, Ministry of Education, Southwest Medical University, Luzhou, Sichuan 646000 China

**Keywords:** Sex hormones, Vascular smooth muscle cells, Phenotypic switching, Diseases

## Abstract

Sex hormones play a pivotal role as endocrine hormones that exert profound effects on the biological characteristics and vascular function of vascular smooth muscle cells (VSMCs). By modulating intracellular signaling pathways, activating nuclear receptors, and regulating gene expression, sex hormones intricately influence the morphology, function, and physiological state of VSMCs, thereby impacting the biological properties of vascular contraction, relaxation, and growth. Increasing evidence suggests that abnormal phenotypic changes in VSMCs contribute to the initiation of vascular diseases, including atherosclerosis. Therefore, understanding the factors governing phenotypic alterations in VSMCs and elucidating the underlying mechanisms can provide crucial insights for refining interventions targeted at vascular diseases. Additionally, the varying levels of different types of sex hormones in the human body, influenced by sex and age, may also affect the phenotypic conversion of VSMCs. This review aims to explore the influence of sex hormones on the phenotypic switching of VSMCs and the development of associated vascular diseases in the human body.

**Graphical Abstract:**

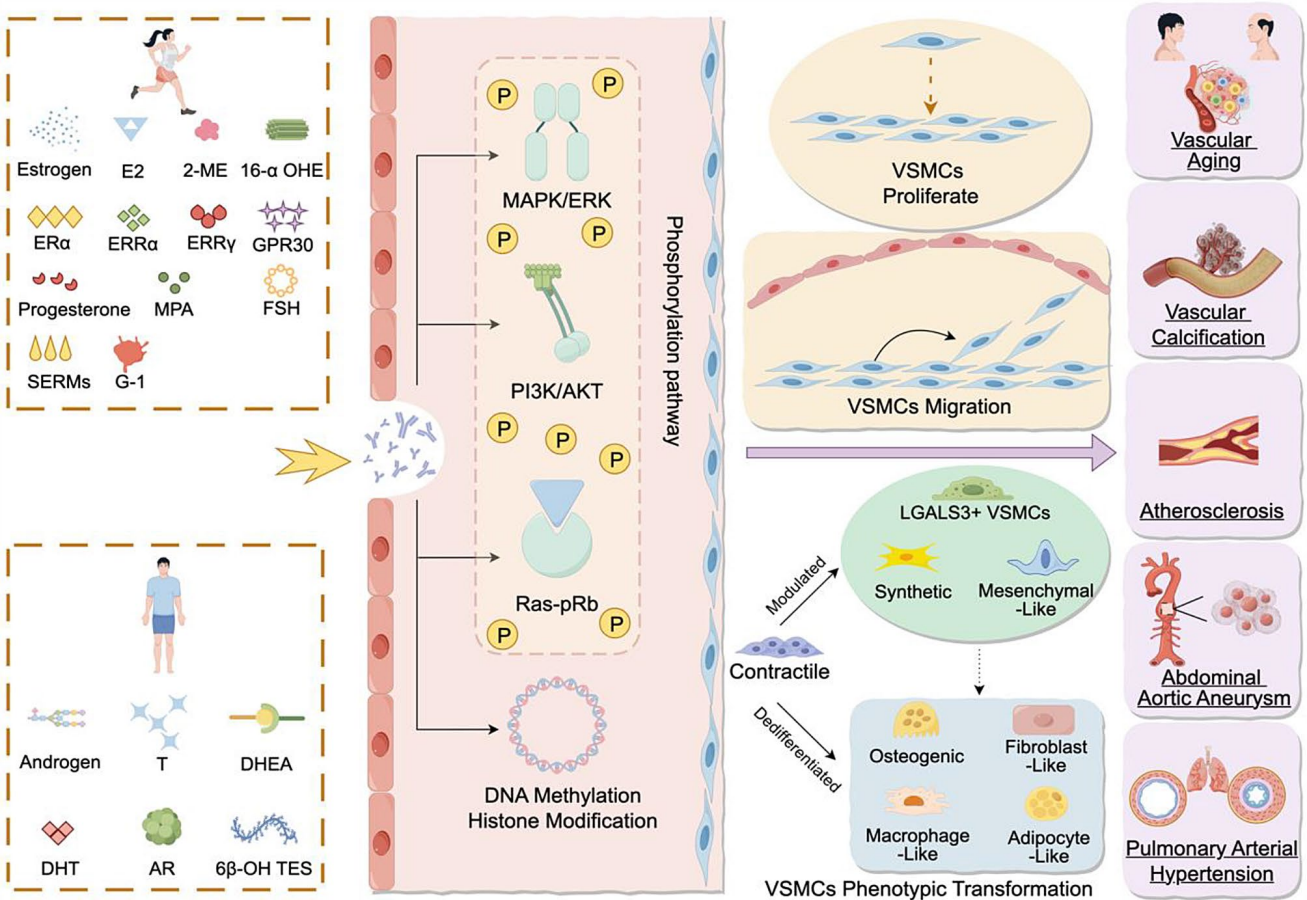

## Introduction

Sex hormones, encompassing androgens secreted by the testes, estrogens produced by the ovaries, and progesterone (Pg) released from the corpus luteum, represent a class of hormones secreted by reproductive and other endocrine glands [1]. These hormones not only dictate sexual characteristics but also exert profound influences on various aspects of human health and metabolism, including the phenotypic switching of VSMCs [2–5].

Under physiological conditions, VSMCs exhibit a contractile phenotype characterized by low proliferative and synthetic activities [3]. However, upon exposure to factors such as hypoxia and inflammation, VSMCs demonstrate their remarkable plasticity by transitioning from this contractile phenotype to alternative phenotypes, a process defined as phenotypic switching of VSMCs. This transformation is accompanied by reduced synthesis of contraction-related proteins, concurrently promoting the upregulation of proinflammatory cytokines and matrix metalloproteinases (MMPs), thereby leading to increased cell migration, proliferation, and secretion [4]. This process plays a pivotal role in the initiation and progression of various cardiovascular diseases (CVDs) [5]. Consequently, a comprehensive understanding of VSMCs phenotypic switching and the promotion of favorable transitions towards beneficial phenotypes paves the way for novel strategies in the prevention and treatment of CVDs.

There is a mutual relationship between sex hormones and the phenotypic switching of VSMCs, which can impact vascular physiology and disease development. Studies have demonstrated that estrogen has a protective effect on VSMCs, as it can suppress their proliferation, migration, and phenotypic switching process [6–8]. This may be related to estrogen’s regulation of intracellular signaling pathways, such as through activation of phosphoinositide 3-kinase/serine/threonine kinase (PI3K/AKT) and extracellular regulated protein kinases 1/2 (ERK1/2) pathways [9]. In contrast, the effects of testosterone (T) on VSMCs are more complex. T can promote VSMCs proliferation and phenotypic transition, increasing the thickness of the vascular wall muscle layer [10]. This may involve T’s regulation of cell cycle proteins and related signaling pathways to influence cell proliferation and differentiation. However, the phenomenon described here only scratches the surface of the effects of sex hormones. In recent years, some scholars have discovered that sex hormones seem to possess dual effects, as they can promote or inhibit VSMCs proliferation, migration, and phenotypic conversion under different conditions [8, 11, 12]. We delve into this topic with greater detail in the following sections.

In summary, sex hormones play an important role in regulating phenotypic switching of VSMCs, but their specific effects are regulated by multiple factors, such as hormone levels, receptor expression, and the interplay of intracellular signaling pathways [8]. These regulatory processes are crucial for maintaining vascular structure and function, as well as for impacting the development of vascular diseases such as atherosclerosis and hypertension. This review thoroughly explores the relationship between sex hormones and phenotypic switching of VSMCs, aiming to provide deeper insights into vascular physiology and disease development mechanisms, and to offer new ideas and strategies for the treatment and prevention of related diseases.

## Physiological function of vascular smooth muscle

The healthy arteries are composed of three-layered ring structures: the intima consisting of a monolayer of endothelial cells, the media containing multiple layers of VSMCs and an elastic fiber layer in between, while the adventitia comprises fibroblasts, adipocytes, connective tissue, and extracellular matrix(ECM) [5].

Mature VSMCs, distinct from their terminally differentiated counterparts in skeletal and cardiac muscle, exhibit remarkable plasticity. Upon differentiation, VSMCs adopt a characteristic fusiform, stable contractile phenotype and specifically express an array of contractile markers, including but not limited to alpha-smooth muscle actin (α-SMA), myosin heavy chain 11 (MYH11), transgelin (TAGLN), smoothelin, and calponin 1 (CNN1), as documented in references [13–15]. However, in response to environmental changes or vascular disease risk factors, such as hypercholesterolemia, hypertension, hyperglycemia, aging, and smoking, VSMCs demonstrate adaptive phenotypic transitions, shifting from a contractile to diverse alternative phenotypes. This process is accompanied by upregulated expression of signaling molecules intimately associated with cell growth, migration, fibrosis, and inflammation, for instance, osteopontin (OPN), desmin, and vimentin, as reported in [16].

**Fig. 1:**
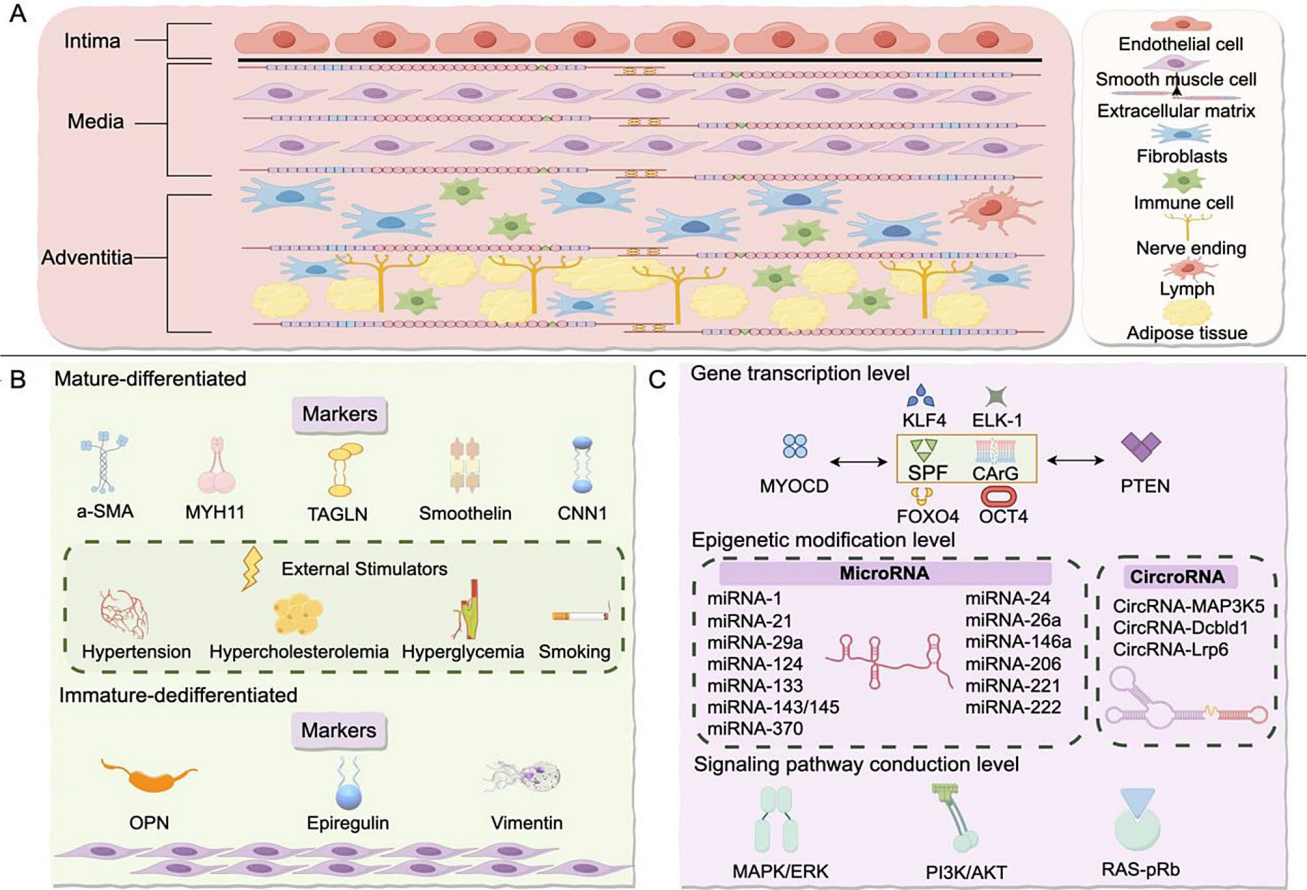
**A**: Vascular layering and cellular distribution within blood vessels, with VSMCs located in the tunica media. **B**: Differentiation and dedifferentiation markers of VSMCs. **C**: Mechanisms of VSMC phenotypic modulation

The mechanisms underlying VSMCs phenotypic modulation are intricate and are orchestrated by intricate regulatory networks involving gene transcription, epigenetic modifications, and signaling transduction pathways. At the transcriptional level, serum response factor (SRF) and its core cofactor, myocardin (MYOCD), constitute the cornerstone of VSMCs phenotypic regulation. By specifically binding to CArG box sequences within 5’ promoters and intronic regions, SRF activates the expression of VSMCs-specific contractile genes [17]. Furthermore, PTEN, a downstream effector of SRF, enhances SRF’s ability to engage critical promoter elements of VSMCs contractile genes through their interaction, a mechanism thoroughly explored in [18, 19]. Additionally, Krüppel-like factor 4 (KLF4) [20], forkhead box O4 [21], transcription factor ELK-1 [22] and octamer-binding transcription factor 4 [23] play indispensable roles in transcriptional regulation. In the realm of epigenetic modifications, MicroRNAs (miRNAs) profoundly influence VSMCs phenotypic transitions through post-transcriptional mechanisms. Specifically, miRNAs such as miR-1, miR-21, miR-143/145, miR-133, miR-124, miR-370, miR-24, and miR-29a have been shown to drive VSMCs differentiation [24–26], whereas miR-24, miR-26a, miR-146a, miR-206, miR-221, and miR-222 facilitate dedifferentiation and proliferation post-vascular injury [26–28]. Moreover, emerging studies have revealed the involvement of non-coding RNAs, including circMAP3K5, circDcbld1, and circLrp6, in regulating VSMCs phenotypic modulation [29–31]. At the signaling pathway level, multiple signaling cascades converge to orchestrate VSMCs phenotypic transitions. Notably, Ras/Raf/MEK/ERK, GSK3β, and β-catenin signaling pathways promote VSMCs phenotypic modulation, whereas Rho-actin, TGF-β, and PI3K/Akt/mTOR signaling pathways exert inhibitory effects [32]. This intricate regulatory network ensures the functional adaptability and phenotypic plasticity of VSMCs under both physiological and pathological conditions. (Fig. 1)

## Phenotypic switching in VSMCs: a comprehensive classification

**Fig. 2:**
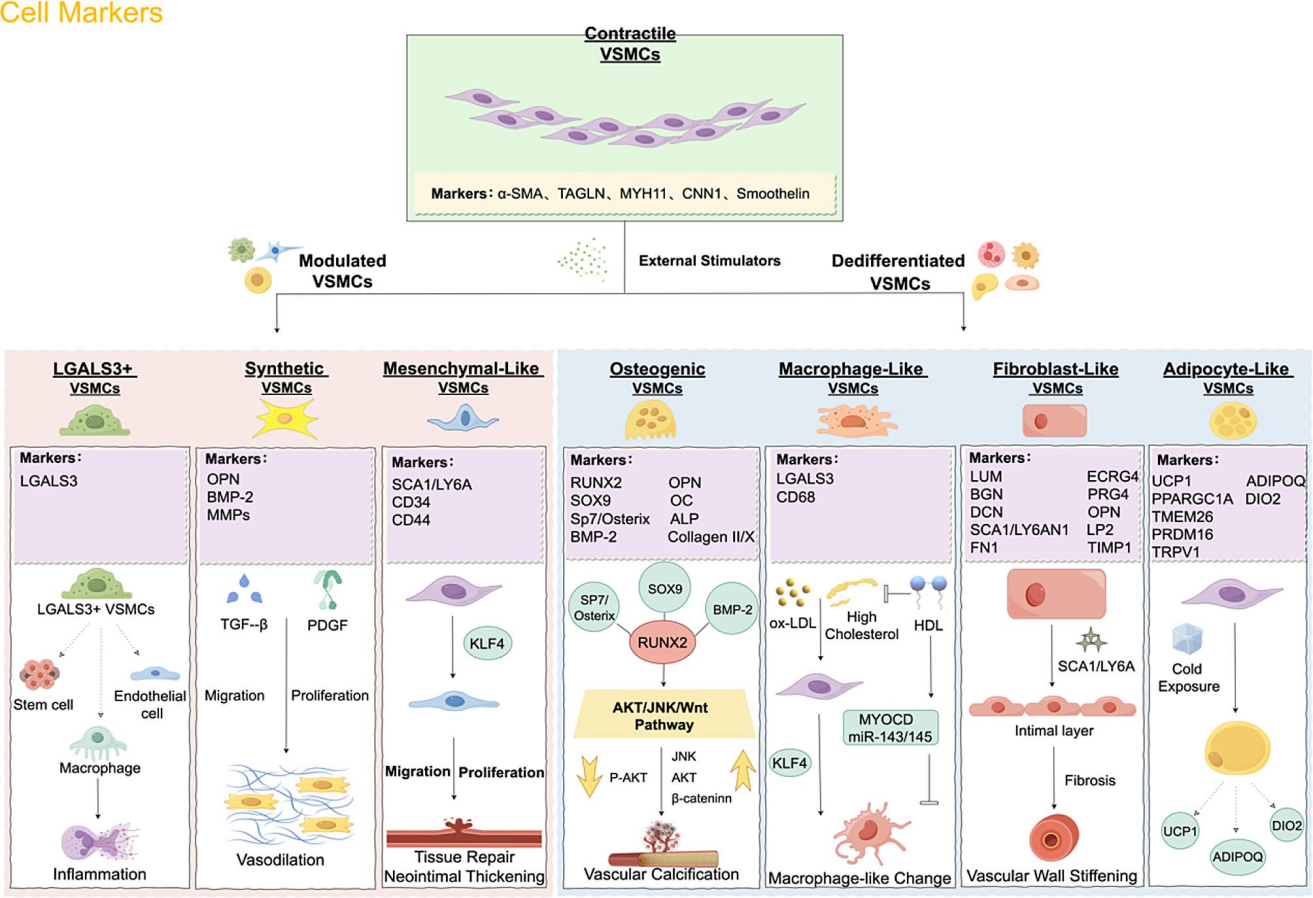
Phenotypic classification and characteristics of VSMCs. Under external stimuli, differentiated VSMCs show reduced expression of contraction markers, giving rise to modulatory and dedifferentiated VSMCs, which express corresponding phenotypic markers. This process influences VSMC proliferation and migration, contributing to the onset and progression of diseases

From a historical perspective, VSMCs have traditionally been thought to undergo a dichotomous phenotypic transition from their contractile state to a so-called “synthetic” state. This implies their responsiveness to various stimuli, switching to a synthetic state and reverting to a contractile state once stimulation ceases. Thus, the contractile and synthetic phenotypes were previously considered the “ideal” phenotypes [33]. However, concerted efforts by numerous scientists have now established that under specific conditions, contractile VSMCs can transition into diverse phenotypes, including intermediate, fibroblast-like, macrophage-like, osteogenic, and adipocyte-like VSMCs [34]. Consequently, the understanding of VSMCs phenotypes has evolved from a simple dichotomous model (contractile and synthetic) to a more complex polymorphic model. Recent research employing single-cell RNA sequencing(scRNA-seq) has revealed a “transitional phase” between contractile VSMCs and their derived cell types, termed modulated VSMCs, which have been implicated in the pathogenesis of atherosclerotic lesions [23, 35]. (Fig. 2)

### Modulated VSMCs

Currently, the characterization of modulated VSMCs predominantly focuses on three categories.

#### Pro-inflammatory macrophage-like phenotype characterized by Galectin-3 (LGALS3) expression

LGALS3 was initially believed to be a characteristic of VSMCs transitioning towards a terminally differentiated macrophage-like state, particularly in lesion cores [20, 36–38]. It was discovered by Alencar et al [23] to be transcriptionally activated in over 60% of VSMCs within atherosclerotic lesions in Myh11-DreERT2-Lgals3-Cre double recombinase Apolipoprotein E (APOE) mice models. Contrary to expectations, these LGALS3 + VSMCs did not differentiate into macrophages but instead displayed at least 4–5 distinct transcriptional profiles, ultimately leading to inflammatory or osteogenic cell states. This subset of LGALS3 + VSMCs exhibited similarities to the fibroblast-like cells previously characterized by Wirka et al [39]. Furthermore, Pan et al [35]. observed in atherosclerotic lesions in both humans and mice that LGALS3 + VSMCs have the ability to differentiate into various cell types, including intermediate cells expressing markers for stem cells, endothelial cells (ECs), and macrophages. Similarly, Hartmann et al [40]. utilized scRNA-seq analysis of lesioned regions in ApoE-deficient mice with SMC-specific deletion of Has3 to identify a notable population of LGALS3-expressing VSMCs. Notably, the emergence of this population has led to advantageous alterations within the lesions. These results collectively confirm LGALS3 as an indicator of the shift from contractile VSMCs to modulatory phenotypes.

#### Synthetic VSMCs

Under stimuli such as hypoxia, inflammation, and mechanical changes, VSMCs can undergo dedifferentiation and transition into a synthetic phenotype [41, 42]. The synthetic VSMCs exhibit a flattened fibroblast-like morphology with a significant increase in cell volume and a decrease in the content of myofilaments and structural proteins in the cytoplasm [43]. Functionally, the contractile ability of synthetic VSMCs weakens, while their proliferation, migration, and secretion capabilities significantly enhance [43]. At the molecular level, the expression of contractile markers is reduced in synthetic VSMCs, whereas the expression of secretion-related molecules, such as bone morphogenetic protein 2 (BMP-2), OPN, MMPs, and various inflammation-related cytokines, is significantly increased [43, 44].

The main function of synthetic VSMCs is to participate in the process of vascular reconstruction and repair. When the blood vessels are damaged or inflamed, these cells are activated and begin to synthesize and secrete ECM molecules, such as collagen, to repair the damaged vessel wall [45]. In addition, they can also produce and release growth factors, such as platelet-derived growth factor (PDGF) and TGF-β which promote the proliferation and migration of VSMCs, further promoting vascular reconstruction [46].

#### Mesenchymal-like VSMCs

Based on lineage tracing studies, contractile VSMCs can undergo a transition to a mesenchymal phenotype, acquiring proliferative and self-renewal abilities while exhibiting reduced expression of contractile proteins [20, 46, 47]. Researchers have defined spinocerebellar ataxia type 1 (SCA1)-expressing VSMCs as mesenchymal-like VSMCs, functioning similarly to mesenchymal stem cells [15]. In response to external stimulation, VSMCs differentiate from contractile to mesenchymal-like phenotypes through inducible repair and/or proliferation processes. KLF4 is considered a key initiator of this phenotypic switch, regulating cell proliferation and dedifferentiation [20, 32, 43, 48–56]. Induction of KLF4 expression leads to the transformation of VSMCs from a contractile to a mesenchymal-like state, characterized by the expression of mesenchyme markers SCA1/lymphocyte antigen 6 family member A (LY6A), CD34, and CD44 [54, 55]. Mesenchymal-like VSMCs are present in the intimal and adventitial layers of arteries [51, 56, 57] Following injury, mesenchymal-like VSMCs proliferate and migrate towards the media and intima, supporting tissue repair and potentially contributing to neointimal thickening [56, 58–60]. In vitro cultured VSMCs, as well as VSMCs subpopulations derived from carotid artery ligation and atherosclerotic plaques, exhibit increased expression of mesenchymal markers SCA1/LY6A [48]. Recent genetic studies indicate that SCA1/LY6A-positive VSMCs have limited impact on atherosclerotic neointima formation, highlighting their context-dependent and injury-specific nature [61]. Identifying human homologs of SCA1/LY6A may further elucidate the plasticity of mesenchymal-like VSMCs as a source of reparative VSMCs and determine their significance in vascular tissue regeneration in the context of vascular diseases [62].

### Dedifferentiated VSMCs

#### Osteogenic VSMCs

During the process of ossification, hypertrophic chondrocytes transform into osteoblasts by generating specific ECM mineralization substances [63]. In the process of calcification associated with atherosclerosis [64] and multiple sclerosis [65], human VSMCs express type II collagen and exhibit a phenotype similar to chondrocytes, referred to as osteogenic VSMCs.

Characteristics of the osteogenic-like phenotype in VSMCs include the loss of contractile markers such as smooth muscle protein 22-α (SM22α) and actin alpha 2, as well as increased expression of calcification markers, such as the runt-related transcription factor 2 (RUNX2), the sry-box transcription factor 9, the zinc finger transcription factor Sp7/Osterix, and BMP-2 [48, 66–69]. Additionally, other markers such as OPN, osteocalcin, alkaline phosphatase (ALP), collagen type II, and collagen type X are also associated with the osteogenic-like phenotype in VSMCs [66, 70]. Both in vitro and in vivo experiments have demonstrated that RUNX2 plays a driving role in VSMCs osteogenesis, while the sry-box transcription factor 9 binds to RUNX2 and inhibits its function [71]. In osteogenic VSMCs, SP7/ Osterix enhances the expression of the RUNX2 gene through interaction and co-regulation of other genes, which is crucial for downstream osteoblast differentiation and bone formation [72]. BMPs are members of the TGF-β protein family, and BMP-2 has been shown to activate RUNX2 in various cell types, playing a critical role in bone repair [73].

AKT, as well as Jun N-terminal kinase(JNK), regulate the osteogenic process involving RUNX2. In human VSMCs exposed to calcifying media, the phosphorylation level of AKT decreases, while the activity of JNK transiently increases, consistent with the notion that elevated levels of AKT and JNK can prevent vascular calcification [74]. This process also involves Wnt signaling, in osteogenic-like VSMCs, Wnt activates β-catenin, which subsequently enters the nucleus and binds to DNA, enhancing the signaling of SP7/Osterix and BMP-2 to facilitate the osteogenic action of RUNX2 [75].

#### Macrophage-like VSMCs

Recent scRNA-seq studies have demonstrated that contractile VSMCs undergo a mesenchymal-like transition, becoming similar to macrophages, which is associated with the development of atherosclerosis [20, 76, 77].The presence of these macrophage-like cells is indicated by LGALS3 and classical macrophage markers, with LGALS3 and CD68 being the most common ones [46, 78]. Oxidized low-density lipoprotein (oxLDL) and high cholesterol are major metabolic factors underlying the occurrence of macrophage-like VSMCs [79, 80]. Exposure to high cholesterol can induce the transformation of VSMCs into macrophage-like VSMCs, with KLF4 playing a crucial role in this process [81]. OxLDL in the elderly promotes VSMCs to acquire macrophage phenotypes and functions [82]. On the contrary, high-density lipoprotein (HDL) exerts reverse effects by removing cholesterol from cells and transporting it to the liver for clearance of peripheral cholesterol. Under the influence of HDL, VSMCs increase the expression of MYOCD and miR-143/145, leading to a reduction in the macrophage-like VSMCs phenotype [81].

#### Fibroblast-like VSMCs

Recent scRNA-seq studies have revealed that in the context of vascular injury, such as the progression of atherosclerosis or the formation of aortic aneurysms, there is a collective phenotypic modulation of VSMCs [39, 83]. TCF21, a fundamental helix-loop-helix transcription factor, is expressed in epicardial progenitor cells during mouse cardiac development, playing a crucial role in the differentiation of fibroblasts and coronary artery smooth muscle cells [84, 85]. Wirka et al [39] utilized scRNA-seq analysis on SMC-specific TCF21 knockout mice in the aortic root and ascending aorta, demonstrating a significant decrease in phenotypic modulation capability and a notable reduction in fibroblast-like cells within lesion and protective fibrous cap regions. Subsequently, they verified that the expression of TCF21 in atherosclerotic lesions in both human and murine subjects is closely linked to the dedifferentiation of VSMCs towards a fibroblastic phenotype, potentially reducing the likelihood of developing coronary artery disease.

In contrast to VSMCs, fibroblast-like VSMCs express fibroblast-specific markers lumican, biglycan, and decorin, while levels of endogenous contractile markers (TAGLN, CNN1) are significantly reduced [39, 86]. Gene expression profiling revealed four major functions of fibroblast-like VSMCs based on scRNA-seq data: synthesizing the ECM, enhancing cell-matrix adhesion, promoting cell proliferation, and regulating collagen deposition [87]. The latter is considered a hallmark of aortic aneurysm formation, closely associated with aortic fibrosis and stiffening processes [87][.Fibroblast-like VSMCs and mesenchymal-like VSMCs share the characteristic marker SCA1/LY6A [39, 50, 86]. Exogenously derived fibroblast-like VSMCs expressing SCA1/LY6A can even migrate to the intimal layer, thereby promoting fibrotic reactions at that site and contributing to vascular wall stiffening [88]. In addition, cholesterol-induced VSMCs phenotypic switching also involves the development of fibroblast-like VSMCs [52, 54]. This transition is accompanied by upregulation of fibroblast-specific markers, including fibronectin 1, ecrg4 augurin precursor, proteoglycan 4, OPN, lipocalin-2, tissue inhibitor of metalloproteinase 1, biglycan, and decorin [54].

#### Adipocyte-like VSMCs

A scRNA-seq analysis has been the only way to describe the phenotype of adipocyte-like VSMCs [46]. This study, using constitutive or inducible Myh11-driven Cre mouse models, demonstrated the ability of VSMCs to adopt this adipocyte-like phenotype [89]. These adipocyte-like VSMCs, classified as beige adipocytes, are also referred to as inducible brown adipocytes [89]. They express key markers of beige adipocytes, including mitochondrial brown fat uncoupling protein 1, peroxisome proliferator-activated receptor gamma coactivator 1 alpha, transmembrane protein 26, domain-containing protein 16, and temperature-sensitive ion channel transient receptor potential cation channel subfamily V member 1 [89, 90]. Following 7 days of cold exposure, these cells can further mature into brown adipocyte-like VSMCs expressing uncoupling protein 1, adiponectin and type 2 iodothyronine deiodinase [89]. The conversion of contractile VSMCs to adipocyte-like VSMCs within the vascular wall may have consequences; however, due to limited research on this phenotype, further investigations are needed to understand its relevance.

## **The impact of sexual arousal on the phenotypic transformation markers of VSMCs.** (Fig. 3)

**Fig. 3:**
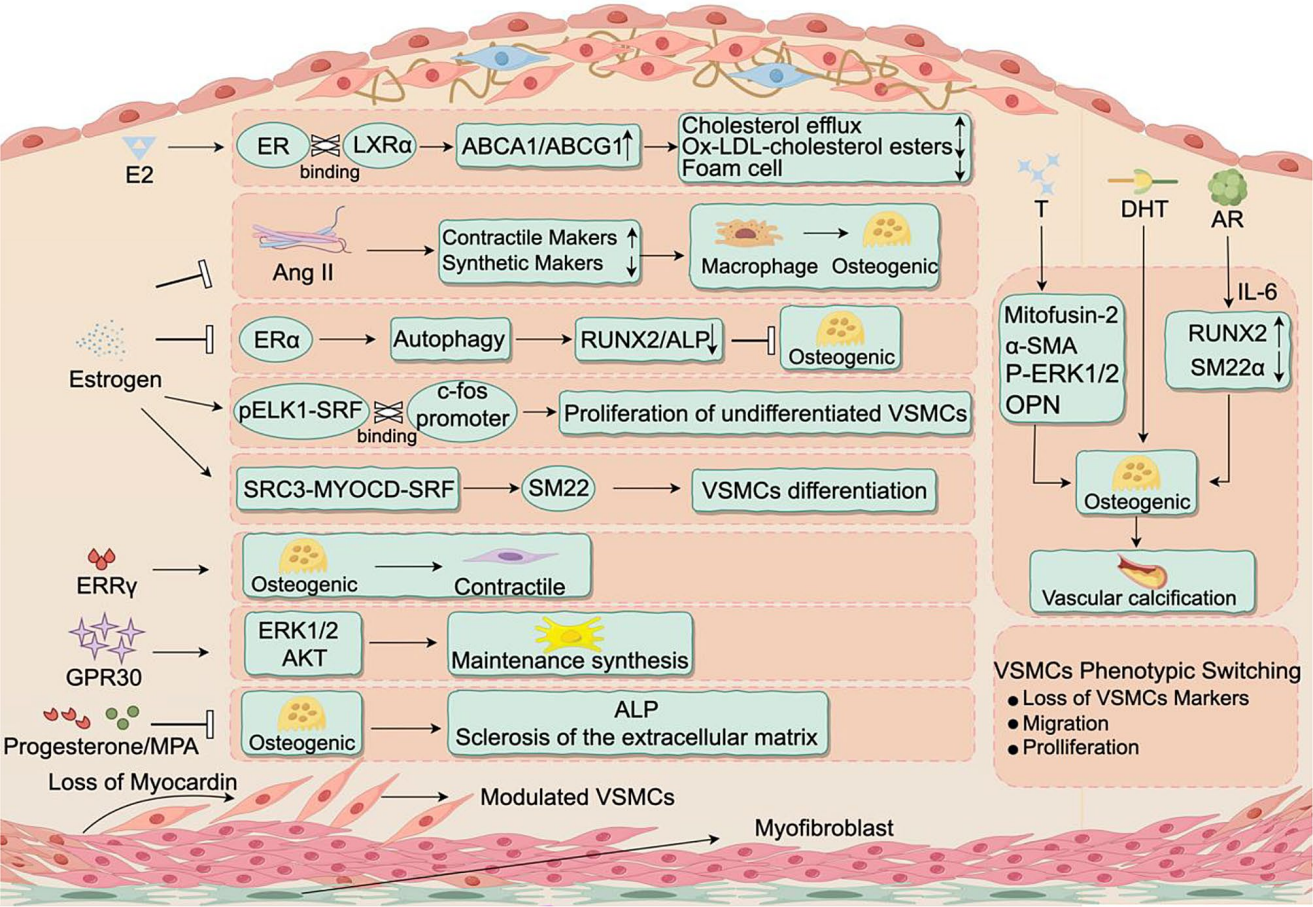
Switches for VSMC phenotypic transformation, estrogen and androgen acting on receptor proteins or other factors influencing the expression of synthetic and contractile markers, mediating VSMCs phenotypic transformation

### Estrogen

Angiotensin II(Ang II)can decrease the expression of smooth muscle contraction markers and increase the expression of synthetic markers. Additionally, it can promote the migration of macrophages and their differentiation into osteoclast-like cells [91]. However, post-treatment with estrogen significantly inhibits the phenotypic transformation of VSMCs and exhibits a concentration dependence [92]. Studies have shown that estradiol (E2) upregulates the expression of ABCA1 and ABCG1 by interacting with estrogen receptor(ER) and liver X receptors α, significantly enhancing cholesterol efflux to APOE and HDL. At the same time, it weakens the accumulation of cholesterol esters in VSMCs induced by oxLDL and reduces foam cell formation derived from VSMCs [93].

Estrogen promotes autophagy in VSMCs through the estrogen receptor alpha(ERα) signaling pathway, reducing the expression of differentiation marker RUNX2 and ALP activity, thereby inhibiting the osteogenic differentiation of VSMCs [94]. Studies have found that activating ERα reduces the expression of differentiation markers in aortic smooth muscle cells [95]. In addition, inhibiting ERα, estrogen receptor beta(ERβ), and/or G-protein-coupled estrogen receptor(GPR30) can reduce the expression of VSMCs calponin [96]. Moreover, MYOCD is a cardiac and smooth muscle cell-specific protein that can activate multiple VSMCs differentiation markers [97]. The interaction of MYOCD with the coactivator steroid receptor coactivator 3 of ER can upregulate the expression of VSMCs differentiation markers [97]. Similarly, MYOCD can induce the transcription and expression of SM22α and α-SMA in rat uterine VSMCs, but when combined with ERα and CArG box, ERα can inhibit this effect [98]. CD34 + VRS/Pcs in the vascular adventitia was first reported by Hu et al [58]. Estrogen has a dual effect on CD34 + VRS/Pcs. Cells in an undifferentiated state are accelerated in their proliferation by pELK1-SRF complex binds to the c-fos promoter and increases transcription. When VSMCs are stimulated, it promotes differentiation through the mechanism of interaction between the MYOCD-SRF complex mediated by coactivator steroid receptor coactivator 3 and the SM22 gene [99].

Estrogen-related receptor γ is a subtype of ER that promotes VSMCs’ dedifferentiation from an osteogenic phenotype during the process of vascular calcification [100]. GPR30 is another type of ER that maintains VSMCs’ synthetic phenotype by activating the ERK1/2 and AKT pathways. It may play a role in the pathogenesis of LDL-induced adventitial calcification and therefore can serve as a new therapeutic target [9].

### Androgen

Recent research has revealed the multifaceted mechanisms through which testosterone regulates the phenotypic transition of VSMCs [10]. In comparison to previous studies, our investigation has demonstrated a more pronounced inhibitory effect of T on oxLDL-induced VSMCs proliferation. Specifically, T induces cell cycle arrest predominantly at the G0/G1 phase and significantly enhances the expression levels of Mitofusin-2 and α-SMA. Furthermore, within a certain dosage range, a slight dose-dependent increase in the expression levels of p-ERK1/2, proliferating cell nuclear antigen, proliferating cell nuclear antigen and OPN was observed [101]. These findings shed light on novel mechanisms by which T governs VSMCs phenotypic regulation.

In inflammatory diseases, androgen receptor (AR) plays a significant role in monocytes/macrophages. By employing AR-silenced culture media to inhibit macrophages, we discovered that it could alleviate inorganic phosphate-induced calcification of human aortic smooth muscle cells (HASMCs) and impede their osteogenic differentiation. This is attributed to the decreased protein expression of osteoblast marker RUNX2 and the increased expression of VSMCs marker SM22α in AR-silenced conditions within the HASMCs [102]. Additionally, we propose that AR’s impact on HASMC calcification may be primarily mediated through the inflammatory cytokine IL-6. Silencing AR in monocytes/macrophages resulted in a significant reduction in IL-6 expression [102]. Immunohistochemical analysis revealed the presence of AR expression in human iliac artery tissue and the calcification media of calcified valves. In vitro studies of VSMCs have revealed that T and dihydrotestosterone (DHT) significantly increase the expression of the mineralization key regulator ALP mRNA. Researchers isolated VSMCs from AR-deficient mice and exposed them to testosterone, observing increased Osterix mRNA expression and upregulation of ALP expression. This finding further underscores that androgen-stimulated VSMCs calcification is mediated through the AR [103].

### Progesterone (Pg)

One of the primary cellular events in vascular calcification is the osteogenic differentiation of VSMCs [104]. Researchers cultured VSMCs in osteogenic media with or without Pg for 21 days. Compared to the control group, treatment with Pg or Medroxyprogesterone Acetate significantly suppressed VSMCs ALP activity and calcium deposition. Further investigations revealed that Pg and Medroxyprogesterone Acetate inhibit VSMCs osteogenic transition through Pg receptor mediation [105]. However, their actions differ when applied to osteoblasts, where Pg promotes maturation and mineralization [105]. This controversial outcome stems from osteoblasts’s potent ability to metabolize Pg into different relevant compounds [106], resulting in progesterone’s dual role: inhibiting calcification in VSMCs while promoting maturation and mineralization in osteoblasts.

## Mechanisms of sex hormone regulation on VSMCs phenotypic switching (Fig. 4)

**Fig. 4:**
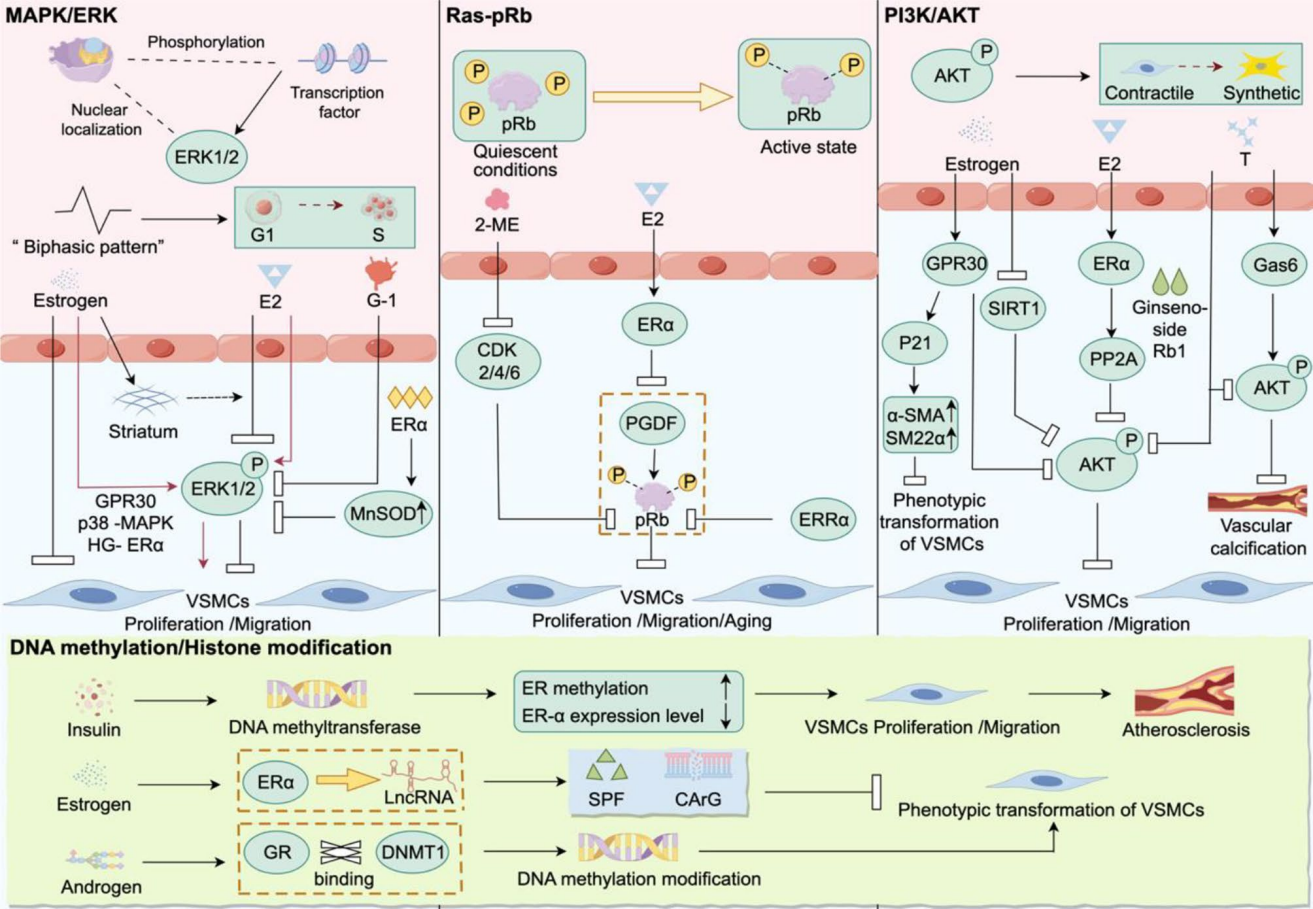
Estrogen and androgen regulate VSMCs proliferation, migration, and participate in disease formation and development. Four classic signaling pathways, MAPK/ERK, Ras-pRb, PI3K/AKT, and DNA methylation/histone modification, enable estrogen and androgen to activate or silence relevant proteins by promoting or inhibiting protein phosphorylation and enhancing methylation, thereby affecting VSMCs proliferation, migration, and phenotypic transformation, driving disease progression

### Receptor-mediated signaling pathway

Estrogens inhibit the proliferation and migration of VSMCs [107, 108], thereby delaying the formation and development of arterial plaques, mainly through the classical MAPK/ERK, Ras-pRb, and PI3K/AKT pathways.

#### MAPK/ERK pathway

ERK1/2 plays a critical role in cell proliferation by exerting its effects through nuclear localization and phosphorylation of transcription factors [109]. Its activity follows a biphasic pattern, characterized by a rapid increase and peak followed by a sustained but lower level during the G phase. This biphasic pattern is crucial for promoting cell progression from G1 to S phase, involving both rapid non-genomic effects and persistent genomic effects [110]. However, whether there are gene expression-independent effects remains to be further investigated. Experimental results in rat VSMCs demonstrate that E2 induces cell proliferation by promoting ERK1/2 phosphorylation [111]. Conversely, when using the GPR30 agonist G-1, it inhibits ERK1/2 phosphorylation in mouse, human, and pig VSMCs, thereby suppressing cell proliferation, migration, and differentiation [112]. Interestingly, other studies have also found that estrogen-induced ERK1/2 phosphorylation may inhibit proliferation and migration of rat aortic VSMCs [9, 113]. This effect appears to be associated with estrogen-induced upregulation of calponin, a calcium-dependent supporting protein [114].

Although these results may seem contradictory, a thorough investigation into the underlying molecular mechanisms may help unravel this puzzle. In fact, in HASMCs, activation of ERα inhibits persistent ERK phosphorylation by upregulating manganese superoxide dismutase, thereby suppressing cell proliferation [115]. Therefore, we can conclude that estrogen induces transient phosphorylation of ERK1/2, allowing VSMCs to enter the cell cycle and express calponin, followed by the inhibition of persistent ERK1/2 phosphorylation in the biphasic pattern.

Specifically, we postulate that estrogen may have a dual regulatory effect on VSMCs function. On one hand, under normal physiological conditions, estrogen inhibits VSMCs proliferation and migration. On the other hand, under pathological circumstances, estrogen can activate the ERK pathway to promote VSMCs proliferation and growth. This activation has also been documented to potentially involve the selective activation or inhibition of ERα through GPR30 [9, 112], p38 MAPK [116], or under high-glucose conditions [115].

#### The Ras-pRb pathway

The retinoblastoma protein (pRb) plays a crucial role in cell proliferation. It is a tumor suppressor protein that regulates the cell cycle by inhibiting E2F transcription factors in a hypophosphorylated state under quiescent conditions [117]. Phosphorylation of pRb is one of the most critical steps for regulating cell cycle progression. Studies have shown that E2 can inhibit PDGF-induced pRb phosphorylation. This effect is mediated by ERα but is independent of ERβ [118]. Additionally, Estrogen-related receptor alpha prevent excessive phosphorylation of pRb, leading to the suppression of rat aortic VSMCs proliferation and neointima formation after vascular injury. These effects may involve the action of the cell cycle-dependent kinase inhibitor p27 Kip1 [119]. By suppressing the activity of CDK2, CDK4/6, 2-methoxyestradiol **(**2-ME**)** can maintain the hypophosphorylated state of pRb, inhibit cell entry into S phase, hinder DNA synthesis, and ultimately suppress VSMCs proliferation and neointimal formation [120]. Furthermore, E2 can delay premature senescence of VSMCs in young female rats by inhibiting the Ras-pRb pathway. However, in aged rats, it may promote senescence. Selective blockade of ER accelerates aging in rats [121]. These findings suggest that estrogen replacement therapy has a protective effect on the vasculature of young women, while the use of estrogen replacement therapy in postmenopausal elderly women may carry potential risks.

#### PI3K/AKT pathway

AKT is considered as an integral component of the PI3K/AKT signaling pathway, which has been extensively studied in various cell types and shown to play a critical role in cell proliferation, survival, and metabolism [122]. In VSMCs, activated AKT can suppress the expression of genes related to muscle contraction while promoting the expression of genes involved in synthesis, thus facilitating the phenotypic transformation of VSMCs. Sex hormones can regulate the phenotypic transition of VSMCs intracellularly, and this process may involve the activation of the PI3K/AKT signaling pathway [123]. Studies have found that in VSMCs isolated from human and pig carotid arteries, estrogen stimulation of GPR30 can inhibit cell proliferation by reducing AKT phosphorylation levels and inducing p21 expression. P21 is a cyclin-dependent kinase inhibitor that induces G1/S phase cell cycle arrest [124]. Additionally, estrogen downregulates SIRT1, promoting VSMCs apoptosis and inhibiting proliferation by inhibiting AKT and ERK phosphorylation [125] Recent research has also revealed that E2 inhibits the proliferation and migration of VSMCs by modulating AKT activity [126].

In HASMCs, testosterone stimulation promotes the expression of growth arrest-specific gene 6 and inhibits vascular calcification through the activation of the AKT signaling pathway [127]. Conversely, research has shown that ginsenoside Rb1 can hinder cell proliferation and calcification under testosterone stimulation by suppressing the AKT signaling pathway [128]. These findings suggest that androgens may have dual effects, highlighting the importance of understanding how to harness the beneficial effects of androgens in future research endeavors.

Furthermore, the PI3K/AKT pathway interacts with other signaling pathways to collectively regulate smooth muscle phenotypic transition. For example, there is reciprocal regulation between the PI3K/AKT pathway and the RhoA/ROCK pathway [129]. This interaction further regulates processes such as cell cytoskeleton reorganization, cell adhesion, and cell migration, which have significant impacts on smooth muscle phenotypic transition.

### DNA methylation and histone modifications

Given that estrogen exerts its effects through binding to its receptors, epigenetic regulation of these receptors may play a crucial role in estrogen-mediated modulation of VSMCs phenotypic switching [130, 131]. In fact, the ER-α promoter is almost unmodified by methylation in normal aortic tissue, whereas it is highly methylated in synthetic VSMCs isolated from human aortas. This high level of methylation contributes significantly to VSMCs phenotypic transition. Other studies have shown that the high degree of methylation of ERα is associated with atherosclerosis and ischemic stroke, and the severity of the disease is positively correlated with the degree of methylation [132–134]. Further research has confirmed this association, revealing that insulin can promote the expression of DNA methyltransferase, leading to an increase in ER methylation [135]. Increased endoplasmic reticulum methylation leads to reduced expression of ERα in VSMCs, resulting in uncontrolled proliferation and promoting the progression of atherosclerosis [135].

In addition, estrogen can also regulate the expression of the SRF gene through ERα-mediated long non-coding RNA, thereby inhibiting smooth muscle phenotypic transition in VSMCs [136]. On the other hand, androgens promote VSMCs phenotypic transition by enhancing the interaction between the glucocorticoid receptor and CpG methyltransferase 1, increasing the level of DNA methylation modification [137].

## The regulatory role of sex hormones in smooth muscle phenotype transition-mediated diseases

### Vascular aging (Fig. 5)

**Fig. 5:**
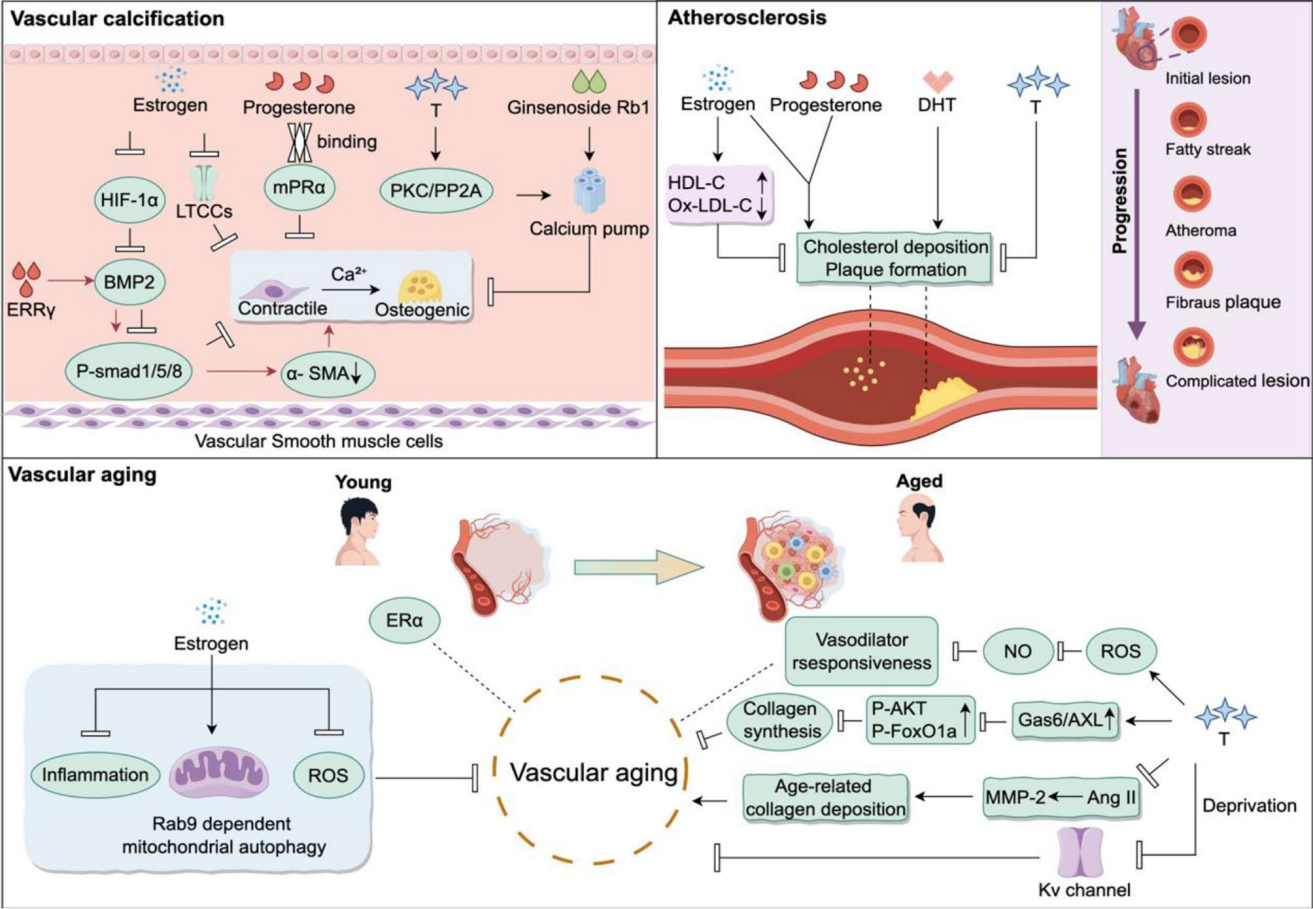
Mechanisms by which estrogen and androgen contribute to vascular aging, vascular calcification, and atherosclerosis onset and development

Vascular aging is characterized by decreased vascular elasticity, increased vascular wall thickness, impaired endothelial function, increased inflammation and arteriosclerosis, and decreased repairability [138]. This process is mainly influenced by the activation of the pro-inflammatory pathway, oxidative stress, cell senescence, and VSMCs proliferation phenotypes. Studies have shown that estrogen slows down the aging process of blood vessels by promoting Rab9-dependent mitochondrial autophagy, reducing oxidative stress and inflammatory reactions, and decreasing vascular wall thickness and stiffness [139].

Previous studies have reported that ERα and ERβ are expressed in the vascular system of both humans and animals, including endothelial and VSMCs [6]. Both ECs and VSMCs are affected by E2, however, their functional roles differ significantly. E2 enhances resistance to injuries in endothelial cells and plays a crucial role in beneficial effects such as enhancing endothelial nitric oxide production, endothelialization, intimal hyperplasia, and atherosclerosis. These actions are primarily mediated through ERα rather than ERβ [140]. In contrast, E2 has minimal pro-angiogenic effects in VSMCs and instead inhibits proliferation and migration by activating VSMCs nuclear ERα-AF1, ultimately preventing neointima formation [141]. In an accelerated aging model, the ERα protein level in the inner layer of the aorta decreases [142]. However, another study found no difference in ERα levels in primary cultures of aortic VSMCs from young (10 weeks) and middle-aged (12 months) mice [143], indicating that further comprehensive research is needed to investigate the age-related changes in ERα expression in VSMCs.

Chen et al. employed T therapy in an Ang II-induced VSMC aging model and found that T delayed VSMCs senescence by reducing MMP2 activity and suppressing collagen synthesis, mediated through the Gas6/AXL and AKT/FOXO1a signaling pathways [144]. Similarly, vascular aging diminishes the expression of calcium-activated potassium channels in VSMCs [145]. Zhou et al [146] discovered that endogenous T deprivation led to downregulation of Kv1.5 expression in rat thoracic aorta VSMCs, while T replacement therapy restored impaired Kv function. This suggests that T may delay vascular aging by activating Kv function in VSMCs. Interestingly, Chignalia et al [147] found that T induces accumulation of reactive oxygen species in VSMCs, reducing NO bioavailability and thereby attenuating vascular relaxation responses. English et al [148] studying arteries of male Wistar rats at different ages, observed that aged vessels are less responsive to T-mediated vasodilation compared to younger counterparts, accompanied by VSMCs hypertrophy and thickening of the smooth muscle layer. The uncertain effects of T on vascular tone regulation may be concentration-dependent, with vasodilation observed only at supraphysiological doses/concentrations [149].

### Vascular calcification (Fig. 5)

Vascular calcification is a complex and dynamic process regulated by multiple factors [150]. It primarily occurs in the media and intima of arteries and involves various types of vascular cells [150]. Among them, VSMCs play a key role in the process of vascular calcification and undergo transformation into osteoblast-like cells. This transformation is characterized by the loss of contractile markers and the acquisition of markers associated with osteoblasts [151, 152].

Studies have shown that estrogen plays an important protective role in maintaining vascular health and inhibiting vascular calcification. It suppresses calcium deposition by regulating cytokines and calcium ion balance. Estrogen therapy can reduce the expression of hypoxia-inducible factor-1α, regulate BMP-2 and downstream signaling pathway Smad1/5/8, thereby reducing vascular calcification in rats [153]. Estrogen-related receptor γ induces the expression of BMP-2, increases the phosphorylation level of intracellular BMP-2 effector protein SMAD1/5/8, and upregulates the expression of osteogenic genes while downregulating the expression of a-SMA [100]. After ovariectomy, the lack of estrogen significantly increases the activity of L-type calcium channels in VSMCs, resulting in an elevation of intracellular calcium ion concentration and promoting vascular calcification [154]. Pg can bind to membrane Pg receptor α, activate the corresponding signaling pathway, and reduce calcium ion concentration in VSMCs [155].

On the other hand, elevated T levels are associated with the development of atherosclerosis and calcification of arteries. T may exert a protective effect on vascular calcification through the activation of protein kinase C signaling pathway and the non-transcriptional pathway of phosphatase, which enhances the activity of calcium pumps and reduces intracellular calcium ion concentration [156]. Moreover, Ginsenoside Rb1, a SARM, can activate the AR signaling pathway, enhance the activity of calcium pumps, reduce calcium ion deposition on the vascular wall, and inhibit vascular calcification [128].

### Atherosclerosis (Fig. 5)

VSMCs are the main cell type found in the subendothelial layer of blood vessels and are responsible for synthesizing matrix molecules such as collagen fibers and elastic fibers, which maintain the structure and stability of the blood vessels. However, under certain conditions, VSMCs can undergo phenotypic switching, resulting in the secretion of large amounts of collagen and MMPs, which may contribute to the formation and progression of atherosclerosis.

Early studies have shown that estrogen has a protective effect against atherosclerosis [157]. It can reduce the oxidation of harmful low-density lipoprotein cholesterol and increase the levels of beneficial high-density lipoprotein cholesterol, thereby reducing the risk of plaque formation [157]. For example, oral estrogen therapy can lower low-density lipoprotein cholesterol levels, increase high-density lipoprotein cholesterol levels, and reduce cholesterol deposition on the vascular wall, ultimately lowering the risk of atherosclerosis [158]. However, the vascular protective effects of oral estrogen are still controversial. The Women’s Health Initiative study in 2002 showed that supplemental estrogen had no cardiovascular benefits and actually increased the risk of cardiovascular complications in postmenopausal women when used in conjunction with progestin [159]. Therefore, understanding the role of estrogen in cardiovascular disease is complex, as it may exert different effects in different tissues and be influenced by individual differences, age, and other factors.

In males, T has a dual nature in its effects. Studies have shown that T can prevent intimal thickening in atherosclerosis. It can protect against neointimal hyperplasia after coronary artery balloon injury and prevent the formation of new intimal plaques in the aorta after ex vivo stripping [160]. However, the DHT can lead to decreased arterial elasticity and arterial sclerosis in female mice, increasing the risk of CVDs [137]. Furthermore, as men age, T levels gradually decrease, which is associated with an increased risk of atherosclerotic CVDs, further emphasizing the complex relationship between sex hormones and atherosclerosis [161].

### Abdominal aortic aneurysm(AAA) (Fig. 6)

**Fig. 6:**
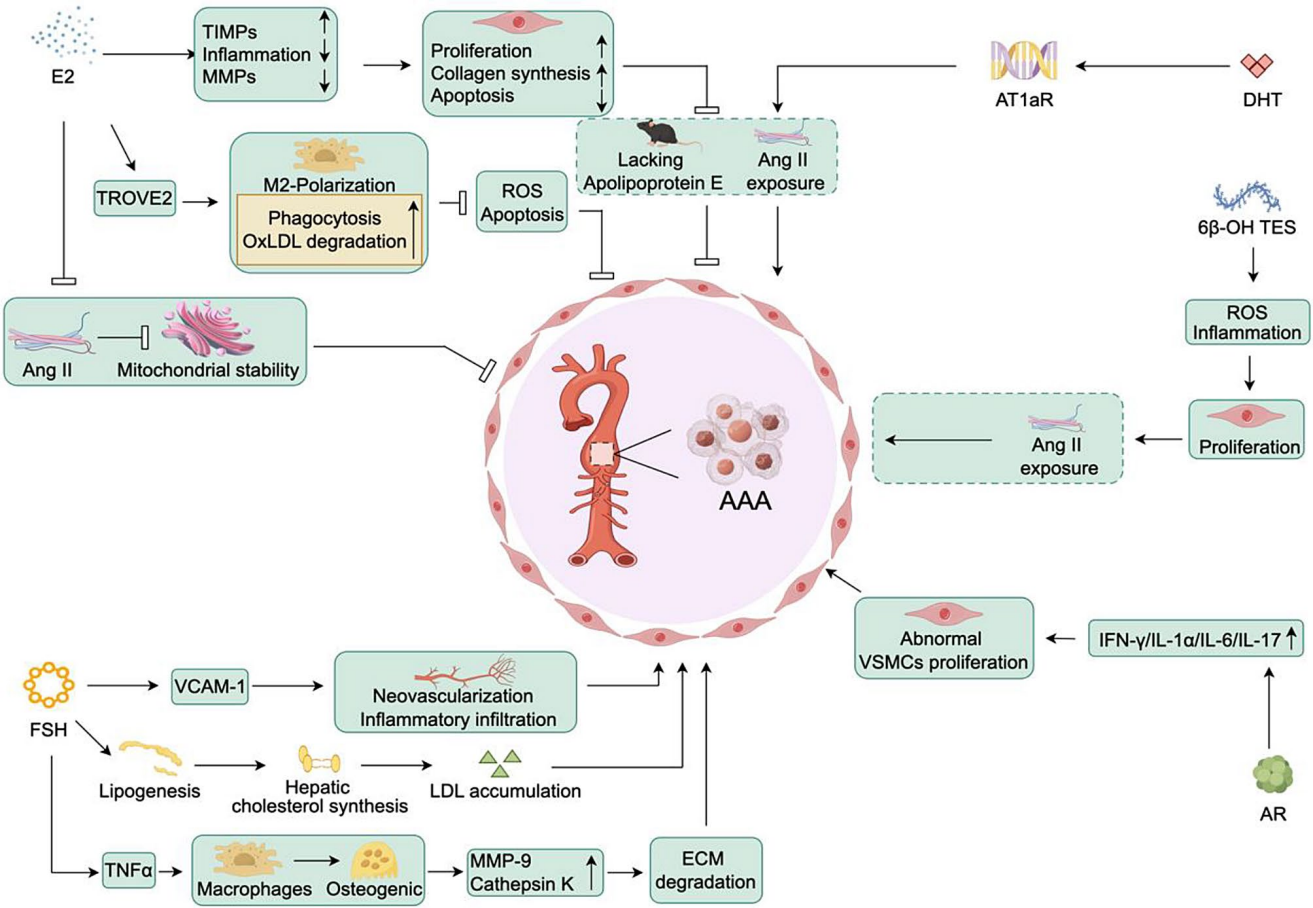
Mechanisms involving estrogen and androgen in abdominal aortic aneurysm

AAA is a pathological dilation of the infrarenal aorta that can lead to aortic wall rupture and catastrophic bleeding, resulting in high mortality rates [162]. At the histopathological level, AAA is characterized by SMC apoptosis, inflammatory cell accumulation, ECM degradation, and oxidative stress occurrence [162, 163]. Previous research has extensively demonstrated the significant role of hormonal factors in the occurrence, progression, and prognosis management of AAA. For instance, in mouse experimental models, estrogen has been shown to reduce inflammation and the expression of MMPs, while simultaneously increasing the expression of tissue inhibitors of metalloproteinases [164]. This leads to a decrease in VSMCs apoptosis, enhanced proliferation and collagen synthesis, ultimately increasing aortic stability and inhibiting the formation and progression of AAA [164]. Estrogen can also regulate the expression of TROVE2, inducing macrophage polarization towards an M2 phenotype. This plays an anti-inflammatory role within the tumor by enhancing macrophage phagocytosis and degradation of oxLDL, reducing ROS and VSMCs apoptosis, thereby inhibiting the occurrence and development of AAA [165]. Furthermore, estrogen can counteract the inhibitory effect of Ang II on mitochondrial membrane potential stability by regulating the expression of mitochondrial membrane channel proteins. This restores mitochondrial function, thereby impeding the development of AAA [166].Similarly, E2 can weaken the formation of Ang II-induced AAA in APOE-deficient mice. This is achieved by reducing the production of inflammatory factors and MMPs, as well as promoting VSMCs proliferation and collagen synthesis [167]. Moreover, in vivo exposure to E2 can suppress MMP2 activity in male VSMCs, leading to a reduction in the size and incidence of male AAA [168].

The phenotypic transition of VSMCs into osteoblast-like cells has been confirmed as a key factor in the occurrence and development of AAA [169]. Estrogen/Pg exert protective effects in premenopausal state, but this safeguard diminishes in postmenopausal state. The decline in estrogen’s protective efficacy has been substantiated across multiple laboratories, giving rise to the “timing hypothesis” [170]. As plasma estrogen levels decline and follicle-stimulating hormone (FSH) levels rise during early menopause [171, 172], attention has shifted towards the role of FSH. Tedjawirja et al. [173]. propose that prolonged elevated plasma FSH levels during the menopausal transition may directly and/or indirectly exacerbate the severity of AAA. They hypothesize that FSH stimulation could promote macrophage differentiation into osteoclast-like cells via TNFα, leading to the production of various proteases (e.g., MMP-9, tissue-type plasminogen activator K), thereby degrading aortic ECM and worsening AAA. Furthermore, FSH may enhance vascular cell adhesion molecule-1 expression in ECs, stimulating neovascularization and inflammation, thereby promoting AAA development. Additionally, FSH stimulates adipogenesis, increases hepatic cholesterol synthesis, promotes low-density lipoprotein accumulation, and exacerbates vascular dysfunction. However, these hypotheses require further validation in vivo and in vitro.

Male gender is an unmodifiable risk factor for the development of AAA. Previous studies have shown that exogenous DHT administration increases the abundance of AT1aR mRNA in the abdominal aorta and promotes Ang II-induced AAA in ApoE-deficient mice [174]. In the mouse model of Ang II-induced AAA, endogenous androgen deprivation results in decreased progressive luminal expansion, significantly alleviating the extent of AAA progression, and reducing both the size of the aneurysm and the degree of wall inflammation [175]. Inhibiting the production of 6β-Hydroxytestosterone or blocking its receptor binding can also significantly reduce inflammation and oxidative stress, inhibit VSMCs proliferation, and alleviate Ang II-induced abdominal aortic aneurysm [176]. Furthermore, activation of the AR increases the expression of inflammatory factors IL-1α, interferon - γ, IL-6, and IL-17, promoting abnormal VSMCs proliferation, leading to the formation and progression of AAA [177].

### Pulmonary arterial hypertension(PAH) (Fig. 7)

**Fig. 7:**
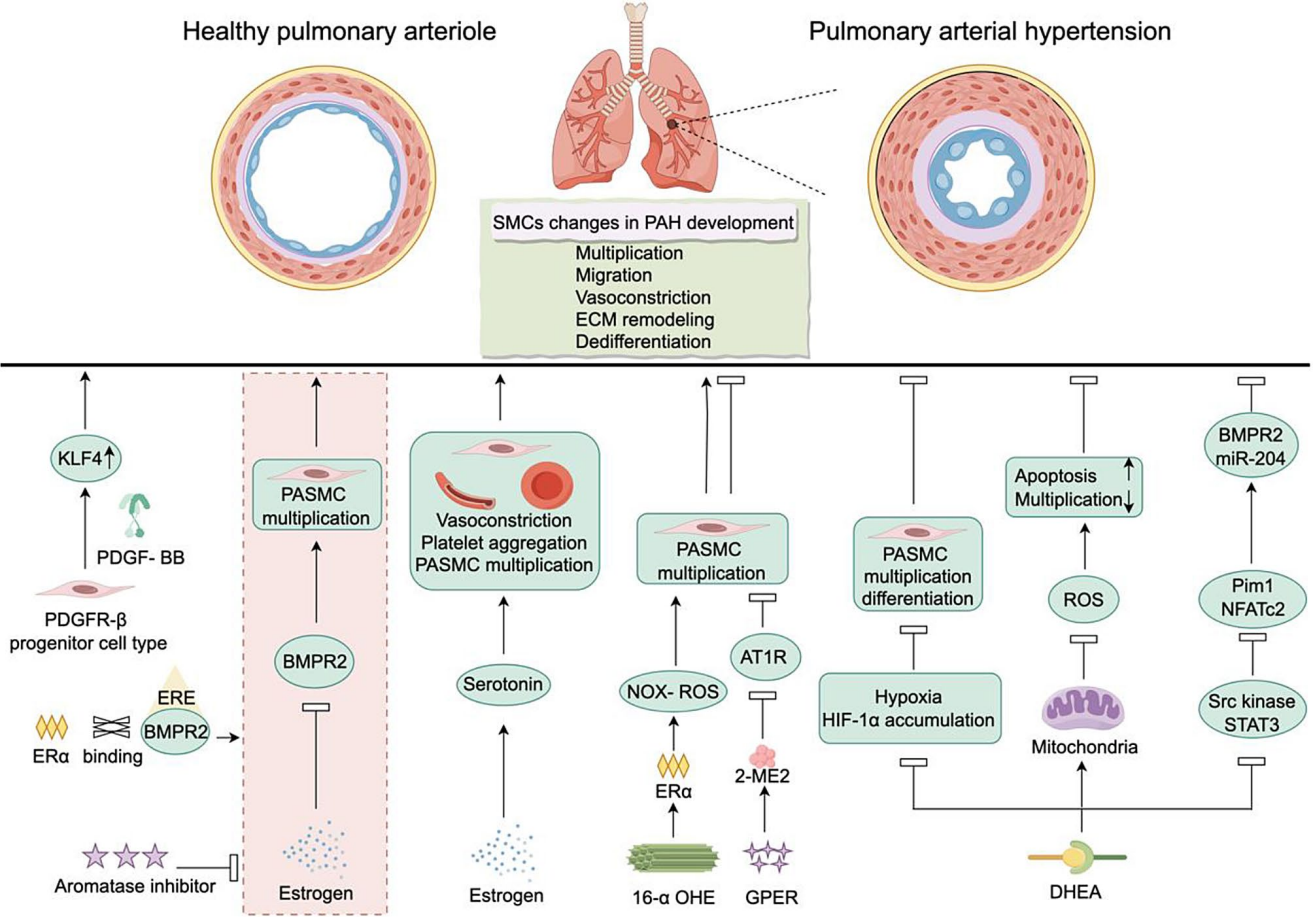
Changes in vascular smooth muscle in pulmonary arterial hypertension, and estrogen and androgen participation in the occurrence and development of pulmonary arterial hypertension by regulating vascular smooth muscle proliferation and migration

The definition of PAH is the formation of complex vascular lesions and vascular remodeling caused by accelerated proliferation of pulmonary ECs, VSMCs, and fibroblast cells [178]. In the early stages, pathologic VSMCs exhibit reduced differentiation, specifically characterized by downregulation of the MYH11 gene expression and upregulation of the PDGFR-β gene expression [179]. Furthermore, studies have also found that PDGFR-β precursor VSMCs upregulate the expression of the KLF4 gene through the PDGF-dependent pathway. To alleviate hypoxia-induced PAH, it is possible to achieve this by depleting KLF4 in VSMCs or depleting PDGF-BB in ECs and macrophages [180–182].

In the past decades, various studies have consistently described the dominant presence of female patients in the population with PAH. Therefore, investigating the unique role of estrogen in PAH is of particular importance. Indeed, mutations in the bone morphogenetic protein receptor type 2 (BMPR2) gene have been identified as one of the most common genetic factors in PAH, and females carrying BMPR2 gene mutations have a higher incidence of the disease [183]. Focusing on this phenomenon, research has found that estrogen can promote excessive proliferation of pulmonary arterial smooth muscle cells (PASMCs) by inhibiting BMPR2 expression [184–186]. This process is enhanced by ERα, which directly binds to the estrogen response elements on the BMPR2 gene promoter [184]. Both mRNA and protein expression levels of female BMPR2 have been observed to be lower in human and murine PASMCs compared to healthy controls and male counterparts [184]. This may partially explain why females are more susceptible to PAH [187]. Estrogen appears to be a “double-edged sword” as under certain conditions, it can also promote BMPR2 gene expression, thereby inhibiting adverse reactions such as proliferation of pulmonary arterial ECs, pulmonary vascular narrowing, and apoptosis of pulmonary arterial ECs, thus protecting right ventricular function [188]. Additionally, estrogen may exert its effects by upregulating the activity of other important mediators in PAH, such as the serotonin signaling pathway, which plays a critical role in the pathological processes of PAH, including promoting vasoconstriction, increasing platelet aggregation, and stimulating cell proliferation [189].

E2 is the most abundant and biologically active estrogen produced by the ovaries, capable of oxidation at multiple carbon positions including C2, C4, and C16. Hydroxylation at the C16 position leads to the formation of high estrogenic metabolites such as 16α-hydroxyestrone (16α-OHE), whereas hydroxylation at C2 results in 2-ME, which exhibits lower estrogenic activity [190]. Both metabolites have been implicated in the onset and progression of PAH. Elevated levels of 16α-OHE were initially observed in urine from female PAH patients with BMPR2 mutations as early as 2013 [191]. Subsequent clinical studies demonstrated that 16α-OHE significantly induces proliferation of PASMCs in PAH patients [192, 193]. Moreover, 16α-OHE stimulates excessive reactive oxygen species generation through NADPH oxidase induced by ERα, leading to oxidative inactivation of protein tyrosine phosphatase and abnormal PASMC proliferation via p38 mitogen-activated protein kinase signaling and cell cycle dysregulatio [194]. Conversely, in contrast to the detrimental effects of 16α-OHE, 2-ME has shown protective effects against PAH. For instance, GPR30 induces 2-ME to inhibit AT1R signaling, thereby suppressing abnormal proliferation in primary rat aortic VSMCs [195] and rat hepatic epithelial cell line [196]. These findings suggest that different estrogen metabolic pathways may exert varying impacts on PAH development.

In PAH, Dehydroepiandrosterone (DHEA) has also been described to have multifunctional protective effects. In human PASMCs, DHEA can exert antioxidant function by regulating mitochondria, thereby reducing proliferation and promoting apoptosis [197]. Consistent with this, DHEA reverses pulmonary artery remodeling through various mechanisms. For example, in an in vitro culture model of hypoxic PASMCs, DHEA treatment has been found to inhibit the accumulation of hypoxia-inducible factor-1α, alleviate proliferation and differentiation of PASMCs, and prevent the formation and progression of PAH [198]. DHEA can downregulate the expression of Src kinase and STAT3 in PASMCs from PAH patients, inhibit their downstream targets Pim1 and NFATc2 expression/activation, restore BMPR2 and miR-204, thereby alleviating adverse reactions such as pulmonary vascular narrowing and increased pulmonary vascular resistance [199].

## Discussion

Sex hormones play a crucial role in regulating phenotypic transformation of VSMCs, involving multiple regulatory aspects. This study represents the first demonstration of how sex hormones influence the morphology, function, and physiological state of VSMCs through modulation of gene expression, signal transduction, and cytokines. Firstly, in terms of gene expression regulation, sex hormones bind with nuclear receptors (such as ERα and ERβ), forming receptor-hormone complexes that bind to hormone response elements on the genomic DNA, thereby influencing gene transcription and expression. These genes include key regulatory factors such as α-SMA and MYH11, which have significant effects on the phenotypic characteristics of VSMCs. Secondly, in terms of signal transduction pathways, sex hormones can regulate VSMCs phenotypic switching by activating or inhibiting Some important signaling pathways include the MAPK/ERK pathway, PI3K/AKT pathway, and Wnt/β-catenin pathway, among others. Moreover, sex hormones can also regulate the synthesis and release of various cytokines, which in turn influence the phenotypic switching of VSMCs. For instance, AR can block IL-6 production, which is involved in the process of VSMCs phenotypic switching.

The current investigation of VSMCs phenotypic transitions and the impact of sex hormones on these transitions is still in its early stages, necessitating further research. While the classification of VSMCs phenotypes has advanced to include modulated and dedifferentiated types, the understanding of modulated VSMCs lags behind that of types such as macrophage-like or osteogenic VSMCs, with limited depth in our comprehension. It is currently uncertain whether LGALS3 + VSMCs possess distinct functions in pathological conditions or if alternative biomarkers exist to indicate “transitional stage VSMCs”. Additionally, the presence of adipocyte-like VSMCs within the dedifferentiated classification has only been documented in a single study, with uncertainties surrounding their functions, the reasons for VSMCs undergoing adipocyte-like transformations, and the precise mechanisms involved.

Moreover, recent investigations into VSMCs phenotypes have validated phenotypic shifts in both arterial and venous VSMCs [95, 200]. Current research on sex hormones and CVDs tends to concentrate on arterial or carotid artery VSMCs, with limited attention given to venous VSMCs [95, 201]. Studies related to venous VSMCs typically focus on venous-related conditions like varicose veins, venous valve damage, and deep venous thrombosis [202–204]. Expanding upon the comprehensive overview provided earlier, the involvement of sex hormones in the phenotypes of arterial VSMCs in vivo or in primary cells and cell lines derived from human or mouse arterial or carotid artery VSMCs, whether in vitro, is of significant interest. It is hypothesized that venous VSMCs may exhibit a comparable significance, given the current evidence indicating that sex hormones prompt venous VSMCs to shift towards a synthetic phenotype [9]. Nevertheless, additional research is needed to determine if they have the potential to transition to alternative phenotypes. The deficiencies in these investigations highlight the extensive journey required to fully comprehend VSMCs phenotypic differentiation.

Through our research, we have partially elucidated the impact of sex hormones on VSMCs phenotype. However, the diverse mechanisms of action of sex hormones and their various receptor types pose complexities. Are the roles of different sex hormones in regulating VSMCs phenotype transition universally applicable? What are the precise signaling pathways and molecular mechanisms through receptors such as ERα, ERβ, and GPR30? Besides these pathways, are there other mechanisms influencing VSMCs phenotype transition? Our current investigations are primarily based on in vitro models, whereas in vivo environments are inherently more intricate. For instance, do significant fluctuations in sex hormone levels during physiological states like aging, puberty, pregnancy, and menopause affect VSMCs phenotype transition? Furthermore, throughout the lifespan, disparities exist in circulating sex hormone levels and types between males and females. How might these gender differences impact VSMC phenotype transition? Additionally, reproductive endocrine disorders have become a focal point of study; women affected by these disorders exhibit disrupted sex hormone expression, with existing literature indicating an increased risk of CVDs among such patients [205]. Do VSMCs phenotypes change in these patients, and if so, what specific alterations occur? Addressing these questions is poised to advance our understanding of VSMCs phenotype transition and the mechanisms of action of sex hormones.

VSMCs exhibit notable phenotypic plasticity, granting them considerable regenerative potential. Therefore, the precise regulation of their phenotype is essential for mitigating the substantial health impact of CVDs. Due to the varied functions of distinct VSMCs phenotypes in different cardiovascular conditions, a comprehensive comprehension of the functional characteristics and specific mechanisms governing VSMCs phenotype transitions is increasingly imperative. This not only assists in identifying potential advantageous aspects of diseases but also enables the advancement of novel disease intervention approaches.

## Conclusion

Through a systematic examination of recent studies on sex hormones and VSMCs phenotype modulation, we have elucidated the crucial role of sex hormones in governing VSMCs phenotype switching and consequent disease advancement. We provide a comprehensive overview of the diverse mechanisms by which sex hormones impact VSMCs phenotype transition. Although there are still many unanswered questions, our research has established a foundation for the discovery of new regulatory factors, signaling pathways, and potential targets related to the modulation of VSMCs phenotype. This offers promising avenues for further exploration and the development of novel therapeutic interventions.

## Data Availability

Not applicable.

## Abbreviations

VSMCs: Vascular Smooth Muscle Cells
Pg: Progesterone
MMPs: Matrix Metalloproteinases
CVDs: Cardiovascular Diseases
PI3K/AKT: Phosphoinositide 3-kinase/serine/threonine kinase
ERK1/2: Extracellular Regulated Protein Kinases 1/2
T: Testosterone
ECM: Extracellular Matrix
α-SMA: Alpha-Smooth Muscle Actin
MYH11: Myosin Heavy Chain 11
TAGLN: Transgelin
CNN1: Calponin 1
OPN: Osteopontin
SRF: Serum Response Factor
MYOCD: Myocardin
KLF4: Krüppel-Like Factor 4
miRNAs: MicroRNAs
scRNA-seq: Single-cell RNA sequencing
LGALS3: Galectin-3
APOE: Apolipoprotein E
ECs: Endothelial Cells
BMP-2: Bone Morphogenetic Protein 2
PDGF: Platelet-Derived Growth Factor
SCA1: Spinocerebellar Ataxia Type 1
LY6A: Lymphocyte Antigen 6 Family Member A
SM22α: Smooth Muscle Protein 22-α
RUNX2: Runt-Related Transcription Factor 2
ALP: Alkaline Phosphatase
JNK: Jun N-terminal Kinase
oxLDL: Oxidized Low-density Lipoprotein
HDL: High-Density Lipoprotein
Ang II: Angiotensin II
E2: Estradiol
ER: Estrogen Receptor
ERα: Estrogen Receptor alpha
ERβ: Estrogen Receptor beta
GPR30: G-Protein-coupled estrogen Receptor
AR: Androgen Receptor
HASMCs: Human Aortic Smooth Muscle Cells
DHT: Dihydrotestosterone
pRb: Retinoblastoma Protein
2-ME: 2-methoxyestradiol
AAA: Abdominal Aortic Aneurysm
PAH: Pulmonary Arterial Hypertension
BMPR2: Bone Morphogenetic Protein Receptor type 2
PASMCs: Pulmonary Arterial Smooth Muscle Cells
16α-OHE: 16α-hydroxyestrone
DHEA: Dehydroepiandrosterone
